# Oral ibuprofen is superior to oral paracetamol for patent ductus arteriosus in very low and extremely low birth weight infants

**DOI:** 10.1097/MD.0000000000016689

**Published:** 2019-08-02

**Authors:** Jinmiao Lu, Qin Li, Lin Zhu, Chao Chen, Zhiping Li

**Affiliations:** a Department of Pharmacy; b Department of Neonatology, Children's Hospital of Fudan University, Shanghai, China.

**Keywords:** acetaminophen, ibuprofen, patent ductus arteriosus, premature infant low birth weight

## Abstract

Paracetamol (acetaminophen) has been proposed as an alternative medication for closing hemodynamically significant patent ductus arteriosus (PDA). However, the clinical outcomes remain inconclusive in very low birth weight (VLBW) and extremely low birth weight (ELBW) infants.

The aim of this study was to compare the efficacy and safety of oral paracetamol and ibuprofen for pharmacological closure of PDA in premature infants from a real-world study.

This retrospective study enrolled 255 preterm infants with birthweights of ≤1.5 kg, and echocardiographically confirmed significant PDA. Subjects were classified into 3 groups: Group I (standard-dose ibuprofen group) received 10 mg/kg oral ibuprofen followed by 5 mg/kg/day for 2 days. Group II (high-dose ibuprofen group) received 10 mg/kg/day oral ibuprofen for 3 days. Group III (paracetamol group) received 15 mg/kg/6 h oral paracetamol for 3 days.

On day 9 after medication start, PDA closure was achieved in 61 (71.7%) patients assigned to the high-dose ibuprofen group, (63.8%) in the standard-dose ibuprofen group, and 33 (37.9%) of those in the oral paracetamol group (*P* <.001). Oral standard-dose ibuprofen was more effective than oral paracetamol (*P* = .001). The ductus closed faster in the high-dose ibuprofen group than in the standard-dose group (median closure time 3.9 ± 1.0 versus 4.4 ± 1.0 days, *P* = .009). Total bilirubin significantly increased in the high-dose ibuprofen group (*P* = .02). No gastrointestinal, renal, or hematological adverse effects were reported. Subgroup analyses indicated paracetamol was minimally effective in ELBW infants (PDA closure 13%).

This study demonstrated that paracetamol may be a poor medical alternative for PDA management in VLBW and ELBW infants. High dosage ibuprofen was associated with a faster clinical improvement and higher rate of PDA closure.

## 1 Introduction

The ductus arteriosus (DA) plays an important role in premature blood circulation, especially in very low birth weight (VLBW) neonates.^[1,2]^ A patent DA (PDA) usually complicates neonatal clinical conditions and increases short and long term risks. In preterm infants, pharmacotherapy with cyclooxygenase (COX) inhibitors such as indomethacin or ibuprofen is the first choice because of its safety and efficacy in treatment of PDA. Surgical closure is the second choice if pharmacological closure is not successful.^[3,4]^ Indomethacin and ibuprofen are both COX inhibitors.^[5]^ Ibuprofen causes fewer side effects than indomethacin and has the same efficacy and safety in closing PDA in preterm infants with respiratory distress syndrome.^[6,7]^ The Chinese Food and Drug Administration requests that indomethacin not be administered in children under 14 years old. Hence, oral ibuprofen has replaced indomethacin for the treatment of infant PDA. The standard recommended oral dose of ibuprofen is 10 mg/kg on the first day, followed by 5 mg/kg for the next 2 days.

Paracetamol has been reported to accelerate the closure of PDA in infants born after 27 weeks of gestation.^[8]^ It was proposed as an alternative medication for closing PDA in recent years.^[9]^ A meta-analysis showed that paracetamol may confer treatment efficacy for closure of PDA comparable to that of ibuprofen with a lower risk of adverse events.^[10]^ However, several studies have shown varied results.^[11]^ Some studies demonstrated a lower PDA closure rate with paracetamol compared to ibuprofen.^[12,13]^ Almost all studies had very small sample sizes, with less than 30 VLBW subjects in each group and even fewer subjects with extremely low birth weight (ELBW). Therefore, the true efficacy of paracetamol in VLBW infants is difficult to determine. It is necessary to observe the efficacy of drugs under real-world conditions. We conducted the present study to compare oral ibuprofen and oral paracetamol for the treatment of PDA in VLBW infants and verify whether paracetamol can replace ibuprofen in clinical settings.

## 2 Materials and methods

### 2.1 Ethical compliance

This retrospective study reviewed records of preterm neonates with PDA in the Children's Hospital of Fudan University in Shanghai from January 2008 to December 2017. All procedures were performed in accordance with ethical standards and the Declaration of Helsinki, and ethical approval was granted by the Institutional Review Board of the hospital (Number FU-ER-LUN-SHEN-109). This retrospective study has been registered at the World Health Organization International Clinical Trials Register Platform (ChiCTR1800017389).

## 3 Patient population

The eligibility criteria included preterm neonates with gestational age less than 30 weeks or birth weight less than 1500 g in the first 2 weeks of life, and with PDA diagnosed with echocardiography and clinical examination. An ELBW infant is defined as 1 with a birth weight of less than 1000 g. The exclusion criteria included maternal use of nephrotoxic medication (e.g., aminoglycosides) within 3 days before delivery, major congenital malformation, proven severe congenital maternal-fetal infection, shock or life-threatening infection, hydrops fetalis, grade 3 or 4 intraventricular hemorrhage, apparent neurological dysfunction (convulsions and coma), and clinical bleeding (apart from isolated pulmonary hemorrhage). Neonates were determined to have symptomatic PDA based on the criteria in previously published studies.^[14,15]^ Hemodynamically significant PDA was defined as signs of PDA plus the presence of cardiomegaly and pulmonary edema on chest radiography, as well as at least 1 of the following: internal ductal diameter ≥ 1.5 mm, left-atrium-to-aortic-root ratio >1.6, unrestrictive pulsatile trans-ductal flow, or reverse or absent diastolic flow in the descending aorta.^[16]^

### 3.1 Treatment protocol

The study included 255 preterm neonates with hemodynamically significant PDA. Neonates were divided into 3 groups. Group I (Standard ibuprofen group, 83 neonates) received an initial dose of 10 mg/kg oral ibuprofen followed by 5 mg/kg/ day for 2 days. Group II (High-dose ibuprofen group, 85 neonates) received 10 mg/kg oral ibuprofen once daily for 3 days. Group III (Paracetamol group, 87 neonates) received 15 mg/kg paracetamol once every 6 hours for 3 days, 60 mg/kg/day. Each group received test medication for 3 days. The medications comprised ibuprofen (Motrin, Johnson & Johnson Pharmaceuticals Ltd.) and paracetamol (Tylenol, Johnson & Johnson Pharmaceuticals Ltd.). All oral drugs were administered by stomach tube infusion. Cardiac ultrasounds were performed when PDA persisted after treatment. If the DA failed to close 6 days after completion of treatment, surgical ligation was considered according to the infant's hemodynamic conditions. Data regarding efficacy and safety were collected and analyzed.

### 3.2 Sample size estimation

Sample size was calculated based on a sample-size estimation formula for comparing proportions with a dichotomous outcome between 2 samples, using an online calculator (www.sample-size.net/). We used a 95% confidence interval level, powered at .8, with a recent meta-analysis showing that a high dose of oral ibuprofen was associated with a significantly higher PDA closure vs a standard dose of ibuprofen (odds ratio [OR] 3.59; absolute difference, 20%).^[17]^ Calculations determined that a minimum sample size of 73 patients per group was required.

### 3.3 Statistical analysis

SPSS version 21 (IBM SPSS Statistics for Windows, Version 21.0. Armonk, NY, IBM Corp) was used to conduct statistical analyses. Categorical data were represented by their respective rates or proportions. T-tests and ANOVA were used to compare means, and a chi-square test was used to compare proportions. The chi-square test was used for categorical variables. Continuous variables were expressed as mean ± standard deviation (SD), and categorical variables were conveyed as frequencies and percentages. The comparison of survival curves utilized Gehan's generalized Wilcoxon Test. *P* values of <.05 were considered statistically significant.

## 4 Results

### 4.1 Efficacy

**Figure 1:**
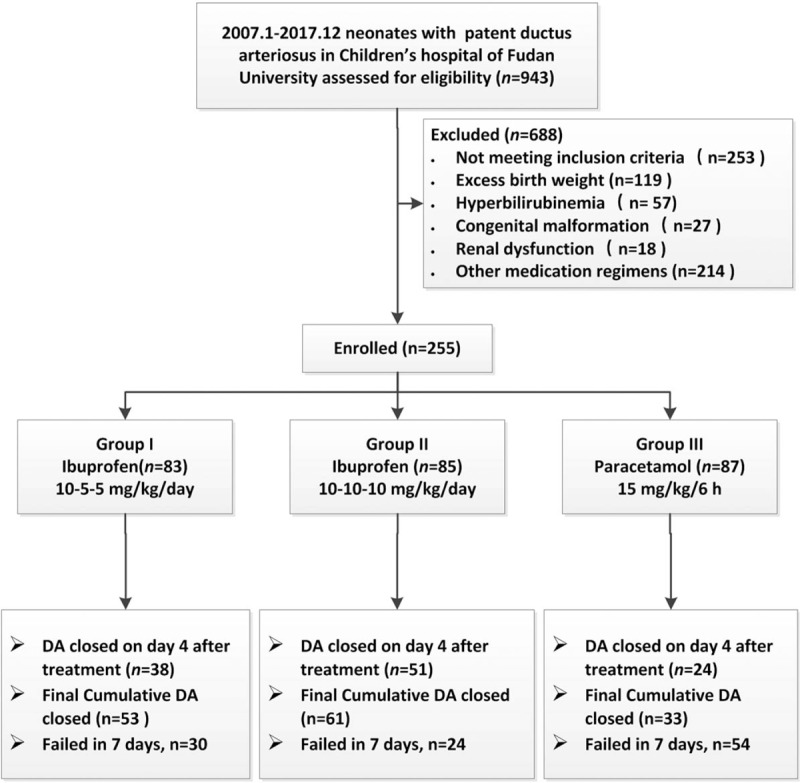
Study assignment and follow-up in each group.

**Figure 2:**
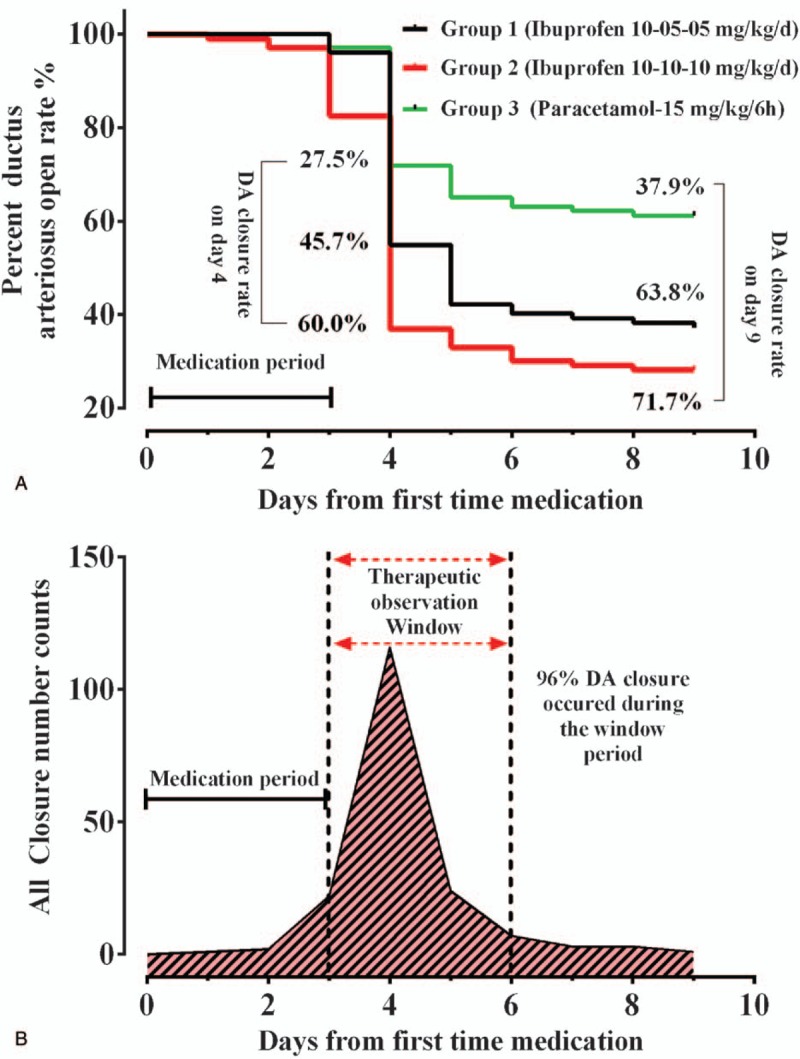
Patent ductus arteriosus closure rate variation with time in different neonate groups. Kaplan−Meier survival curve for ductal closure. Percentage of paracetamol and ibuprofen infants with open ductus up to 9 days after first medication (A). Cumulative calculation of closure number within days after treatment (B).

A total of 943 infants had medically treated DA during the study period, and 255 patients were enrolled. A flow chart of the study according to drug and dosage is illustrated in the STROBE Flow Diagram (Fig. 1). The clinical characteristics and echocardiographic findings of the preterm infants are summarized in Table 1. Demographic and echocardiographic parameters between all groups were not statistically different before treatment. The DA was completely closed in 38 (45.7%) of the patients assigned to the oral ibuprofen groups and 24 (27.5%) from the oral paracetamol group on the first treatment day. The closure rate in the paracetamol group was significantly lower than in the ibuprofen groups (*P* <.01). The cumulative closure rates increased for all groups over 9 days (Fig. 2A). By the end of the 9th day after initiation of treatment, the DA was completely closed for 61 (71.7%) patients in Group II (high dose ibuprofen), 53 (63.8%) in Group I (standard dose ibuprofen), and 33 (37.9%) in Group III (paracetamol). The ductus closed faster in the high dose ibuprofen group than in the standard-dose group (median closure time 3.9 ± 1.0 versus 4.4 ± 1.0 days, *P* = .009). For all patients, 96% of DA closures occurred within 3 days after completion of the treatment protocol. Hence, we concluded that the best time for our treatment evaluation was 3 days after completion of drug administration (Fig. 2B).

**Table 1:**
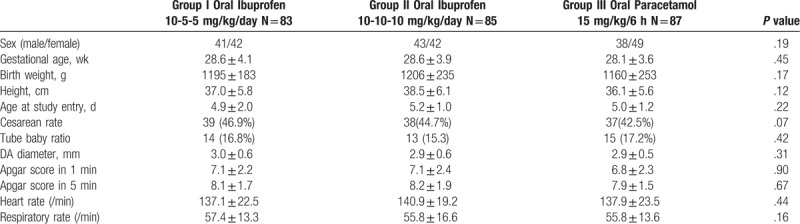
Baseline and demographics of each group.

### 4.2 Adverse events

While renal tolerance is a concern with COX inhibitors, none of the patients exhibited oliguria. No statistically significant difference in adverse outcomes was observed among the 3 groups. Direct bilirubin was not significantly increased from pre-treatment in any group, and no other adverse effects were evident (Table 2). Although a small total bilirubin level increase in the high-dose ibuprofen group (up 18%, *P* = .02) was not clinically significant, the potential risk for liver toxicity in VLBW infants cannot be ignored.

**Table 2:**
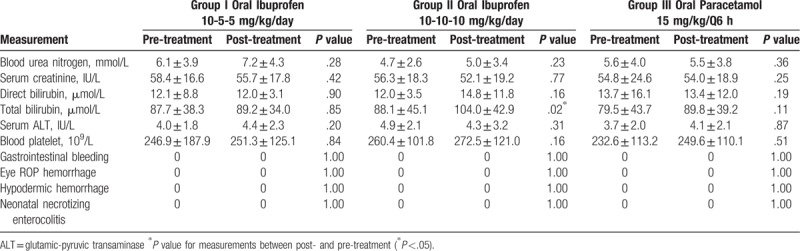
Evaluation of different parameter on days 1 and 9 among different study groups.

### 4.3 Subgroup and sensitivity analyses

In a subgroup analysis of ELBW infants in each treatment group, the low-dose ibuprofen group had a DA closure rate of 44% (7/16), while the high-dose ibuprofen group had a closure rate of 60% (9/15). There was no significant difference in the baseline characteristics of the ELBW subgroups. The paracetamol subgroup had the lowest closure rate of 13% (3/23). On the 1 hand, this result suggests that closing the DA is more difficult with younger gestational age. On the other hand, although paracetamol is very safe in ELBW births, its efficacy is limited. Sensitivity analyses with ELBW infants omitted were used to evaluate the robustness of the results. The DA closure rates of Groups I, II, and III were 70.1%, 74.3%, and 48.4%, respectively. These were consistent with the results of the total study, suggesting no significant bias.

### 4.4 Odds ratios (OR)

We set the paracetamol group as a control group. Absolute differences (%) and relative risks (OR with 95% confidence intervals [CIs]) were compared. The control versus standard-dose ibuprofen group comparison resulted in a 27% absolute difference, OR 2.89, 95% CI [1.55, 5.39], *P* = .0008. The control versus high-dose ibuprofen group comparison yielded a 34% absolute difference, OR 4.36, 95% CI [2.19, 7.89], *P* <.001. In summary, ibuprofen treatment led to substantially greater improvements in DA closure than paracetamol within the 3-day treatment regimes.

## 5 Discussion

Although both oral and intravenous ibuprofen are currently used for treating and preventing PDA, oral therapy is less expensive and may be safer than intravenous therapy in neonates. Several previous studies, including 1 systematic review and meta-analysis, have shown that oral ibuprofen is as effective as intravenous ibuprofen and has fewer side effects.^[18–23]^ Further, 1 trial has specifically shown that oral ibuprofen is more effective than intravenous ibuprofen for ductal closure in VLBW infants.^[24]^ In the long run, preterm infants who were treated with oral ibuprofen for PDA had similar neurological, neurosensory, and cognitive outcomes at 2 years of age to patients who received intravenous ibuprofen.^[25]^

Oral ibuprofen has also been shown to be as efficacious as intravenous indomethacin and there were no differences in safety.^[22]^ Further, in VLBW infants, oral ibuprofen is as effective as intravenous indomethacin for closure of PDA and is associated with significantly less necrotizing enterocolitis and lower rates of elevated creatinine levels.^[18–20]^ The need for postnatal steroid use for chronic lung disease was significantly lower in the oral ibuprofen group than in the Intravenous ibuprofen treatment group in ELBW infants.^[21]^ On the other hand, at least 1 study raised concerns about increased sepsis and bronchopulmonary dysplasia incidence with oral ibuprofen as compared to intravenous ibuprofen.^[23]^ However, the evidence overall supports the use of oral ibuprofen as a safe and effective alternative agent for the treatment of PDA in neonates.

### 5.1 Real world evidence and clinical trials in PDA treatment

Real world evidence (RWE) is the clinical evidence about the usage and potential benefits or risks of a medical product derived from analysis of real-world data. Real world data (RWD) are data relating to patient health status and/or the delivery of health care routinely collected from a variety of sources. In December 2018, the US Food and Drug Administration (FDA) created a framework for evaluating the potential use of RWE to help support the approval of new indications for approved drugs or to help support or satisfy post-approval study requirements.^[26,27]^ This method is especially suitable for clinical research in severe early childhood diseases.

In the clinical real-world practice for our study, only the infants who were confirmed to have PDA by ultrasound on day 3 or later after delivery were administered non-steroidal anti-inflammatory drugs. It is often necessary to observe the DA status for 3 to 5 days before drug administration in real-world clinical practice, because nearly 70% of PDAs will naturally close within 3 days after birth.^[28]^ However, many well-designed clinical trials have prophylactically treated newborns less than 3 days after delivery. Some medications for PDA are even used in the first day after birth. These clinical studies ignore the natural PDA closure rate in newborns, making it difficult to distinguish whether closures were spontaneous or due to the efficacy of drugs and possibly exaggerating the clinical efficacy of drugs.

An epidemiological study found that approximately 1.3% of all newborns were diagnosed with very low birth weight (<1500 g) in the United States, and the number of VLBW infants and sample sizes in previous trials were very small and the results are less than convincing.^[29]^ There are particularly small cohorts in trials with ELBW newborns. In addition, it has historically been very difficult to conduct drug studies in children for a number of ethical reasons.^[30]^ Therefore, it is necessary to observe the efficacy of drugs under real-world conditions.

### 5.2 Efficacy and safety of paracetamol and ibuprofen

All treatment regimens in this study were well-tolerated and considered safe in terms of renal and liver variables and there was no statistically significant difference in major complications (renal tolerance, hyperbilirubinemia, gastrointestinal bleeding, necrotizing enterocolitis (NEC), and retinopathy of prematurity (ROP). In accordance with other trials, there was no significant difference between paracetamol and ibuprofen in neonates. ^[29,31]^

El-Farrash RA,^[32]^ reported that paracetamol and ibuprofen are similarly effective for PDA treatment. In contrast, our results showed that paracetamol is remarkably less effective than ibuprofen (*P* <.05) for the closure of PDA in neonates, and specifically less effective in VLBW or ELBW infants. The reason may be that VLBW newborns are different from newborns with longer gestational age. Similarly, Sallmon H^[33]^ observed that the complete closure rate following paracetamol administration is only 21.1% (4/19) in VLBW infants, in line with the closure rate of 27.5% (24/87) observed after paracetamol treatment in this study. Further, 3 other trials revealed that paracetamol was not as effective as ibuprofen,^[34,35]^ and in a fourth trial PDA closure was only 18% with high-dose intravenous paracetamol in VLBW infants.^[36]^

Hemodynamically significant PDA has many complications for preterm and low birth weight neonates and closure is strongly recommended. The choice of drug used for medical closure of PDA may depend on several factors. For example, the presence of even a grade 1 cerebral hemorrhage may be an indication to use paracetamol rather than ibuprofen. In other cases, a patient may develop a contraindication for ibuprofen during treatment with ibuprofen as the first choice therapy, and then require a change to paracetamol. In any case, existing research has shown no significant difference of hemoglobin levels in paracetamol use compared with other cyclooxygenase inhibitors,^[37,38]^ and no studies have shown a direct protective effect of paracetamol on cerebral hemorrhage.

### 5.3 Paracetamol has less COX-2 binding affinity and effect than ibuprofen

Paracetamol showed no vessel contractile effect on the DA in premature rats while indomethacin showed a dose-dependent effect.^[39]^ As we know, prostaglandin E2 plays a key role in the closure of the DA, and cyclooxygenase-2 contributes to >90% of prostaglandin E2 formation.^[40]^ Strong cyclooxygenase-2 inhibitors like ibuprofen may serve as better alternatives for the neonate than nonselective cyclooxygenase blockers such as paracetamol. The DA reopening rate was shown to be higher in paracetamol treatment than in ibuprofen treatment.^[13]^ Thus, we speculate that the efficacy of paracetamol in VLBW infants is different from the efficacy in older premature or full-term infants. Developmental pharmacological factors may exist behind this phenomenon.^[41]^ In addition, high dosage ibuprofen therapy was more effective with fewer adverse effects than the standard dosage, consistent with our previous clinical findings.^[42]^ What's more, exposure to ibuprofen did not affect neonatal renal function.^[43–45]^ Thus, our findings show that oral ibuprofen is superior to oral paracetamol in VLBW infants (closure rate 63.5% vs 38.8%), and especially in ELBW infants (closure rates 44% vs 13%).

Ibuprofen is only used for 1 course of treatment (3 days) in preterm infants, in order to avoid long-term adverse drug reactions, because non-steroidal anti-inflammatory drugs have a longer metabolic time in preterm infants. Some literature recommends 5 to 7 days or more of paracetamol treatment for PDA, while other literatures recommend only a 3 days course, similar to the administration strategy for ibuprofen.^[46,47]^ This may explain the low closure rate seen with paracetamol treatment in our study. However, no significant research studies show better efficacy with a 7 days treatment compared to a 3 days treatment. Larger prospective studies are needed to explore the negative tendency suggested by our results and to assure safety.

### 5.4 Limitations

One limitation of the study is the lack of a control group and double-blinded evaluations. The present study excluded a placebo control group, and we failed to estimate the spontaneous DA closure rate. In addition, the study was incompletely blinded due to different daily doses, although the outcome assessors were completely blinded to the treatment groups. We suggest further studies for comparison of treatments in a larger sample size of infants with PDA. Additional well-designed studies are also advocated to support the use of paracetamol for DA in the current clinical practice. According to our results, the best window of evaluation of therapeutic efficacy is 3 days after a course of medication.

## 6 Conclusion

In summary, we do not recommend the use of paracetamol for PDA closure in VLBW and ELBW infants. A standard dose of 10 mg/kg of ibuprofen, followed by 5 mg/kg for 2 days, can be safely given to VLBW infants with good efficacy. A higher ibuprofen dosage (10 mg/kg once daily for 3 days) was associated with a higher rate of closure and faster clinical improvement, although caution is suggested with higher doses in VLBW infants due to the risk of toxicity. Oral ibuprofen is the first choice for PDA treatment in VLBW preterm infants. Paracetamol represents an enticing novel therapy due to wide availability, low cost, and an appealing safety profile. However, paracetamol may be a poor medical alternative in PDA management in VLBW infants, as it is non-toxic but ineffective. Ongoing investigation is required to determine the role of paracetamol in PDA treatment algorithms. Pending these results, clinicians must weigh the potential risks and benefits of each therapy for individual neonates considering all available evidence.

## Author contributions

**Investigation:** Jinmiao Lu.

**Methodology:** Lin Zhu.

**Software:** Qin Li.

**Supervision:** Qin Li, Chao Chen, Zhiping Li.

**Writing – original draft:** Jinmiao Lu.

**Writing – review & editing:** Jinmiao Lu.

Zhiping Li orcid: 0000-0001-6194-023X.
